# Scoping Reviews, Systematic Reviews, and Meta-Analysis: Applications in Veterinary Medicine

**DOI:** 10.3389/fvets.2020.00011

**Published:** 2020-01-28

**Authors:** Jan M. Sargeant, Annette M. O'Connor

**Affiliations:** ^1^Department of Population Medicine, University of Guelph, Guelph, ON, Canada; ^2^Department of Veterinary Diagnostic and Production Animal Medicine, Iowa State University College of Veterinary Medicine, Ames, IA, United States

**Keywords:** evidence synthesis, scoping reviews, systematic reviews, meta-analysis, veterinary

## Abstract

Evidence-based decision making is a hallmark of effective veterinary clinical practice. Scoping reviews, systematic reviews, and meta-analyses all are methods intended to provide transparent and replicable ways of summarizing a body of research to address an important clinical or public health issue. As these methods increasingly are being used by researchers and read by practitioners, it is important to understand the distinction between these techniques and to understand what research questions they can, and cannot, address. This review provides an overview of scoping reviews, systematic reviews, and meta-analysis, including a discussion of the method and uses. A sample dataset and coding to conduct a simple meta-analysis in the statistical program R also are provided. Scoping reviews are a descriptive approach, designed to chart the literature around a particular topic. The approach involves an extensive literature search, following by a structured mapping, or charting, of the literature. The results of scoping reviews can help to inform future research by identifying gaps in the existing literature and also can be used to identify areas where there may be a sufficient depth of literature to warrant a systematic review. Systematic reviews are intended to address a specific question by identifying and summarizing all of the available research that has addressed the review question. Questions types that can be addressed by a systematic review include prevalence/incidence questions, and questions related to etiology, intervention efficacy, and diagnostic test accuracy. The systematic review process follows structured steps with multiple reviewers working in parallel to reduce the potential for bias. An extensive literature search is undertaken and, for each relevant study identified by the search, a formal extraction of data, including the effect size, and assessment of the risk of bias is performed. The results from multiple studies can be combined using meta-analysis. Meta-analysis provides a summary effect size, and allows heterogeneity of effect among studies to be quantified and explored. These evidence synthesis approaches can provide scientific input to evidence-based clinical decision-making for veterinarians and regulatory bodies, and also can be useful for identifying gaps in the literature to enhance the efficiency of future research in a topic area.

## Background

Evidence-based decision-making is a hallmark of veterinary clinical practice and veterinary public health. Evidence-based veterinary medicine has evolved from principles of evidence-based medicine developed in the human healthcare literature. The evidence-based medicine approach integrates patient values, clinical expertise, and scientific evidence to make decisions about the clinical care of patients (1, 2). Within this approach, scientific evidence is derived from the results of research studies. However, clinical trials may differ in their inclusion criteria and recruitment, and trials are conducted on a sample of the target population; therefore, the results of a single study represent a random result from a distribution of possible trial results (3, 4). Additionally, there is empirical evidence that the first study on a given topic will have the largest effect size, with diminishing or contradictory effect sizes reported in subsequent studies (3, 5). As a consequence of these concepts, decision-makers should use the body of evidence rather than a single study result, as the unit of concern for making evidence-based decisions. However, it is time consuming for veterinarians and others involved in veterinary decision-making to identify, acquire, appraise, and apply the available literature on a given topic. For instance, a simple search in PubMed using the search string cattle AND (BRD or “bovine respiratory disease”) AND (vaccine or vaccination) resulted in the identification of 286 potentially relevant articles (search conducted Jan 10th 2020). Thus, it is essential both to replicate research and to have a means of combining (synthesizing) the results of multiple studies addressing the same research question.

Evidence synthesis refers to the combination of results from multiple sources. There is a plethora of methodologies for undertaking evidence synthesis for various types of information or types of synthesis questions (6). This paper focuses on two common evidence synthesis tools used in veterinary medicine: scoping reviews and systematic reviews. Meta-analysis, the statistical summarization of results from multiple studies, is the analytical component of a systematic review which can be undertaken when there is a sufficient body of literature identified in the review. Both scoping reviews and systematic reviews are methods to synthesize existing literature by following a series of structured and documented steps, and using methods intended to reduce the risk of bias. However, the two types of reviews answer different research questions. Scoping reviews are a descriptive study design, intended to chart or map the available literature on a given topic. By contrast, systematic reviews answer a specific question, often related to clinical decision-making, with the ideal end product being a summarized effect or effect size across multiple studies or an exploration of sources of heterogeneity (differences among studies in the effect or effect size).

## Methods

### Scoping Reviews

Scoping reviews are used to describe the available literature on a topic (often referred to as charting or mapping). The specific objectives of a scoping review might be to describe the volume and nature of the existing literature in a topic area, to determine the feasibility of conducting a systematic review for a specific review question within a topic area, or to identify gaps in the body of literature on a topic (7, 8). The approach was first described by Arksey and O'Malley (7) and further advanced by Levac et al. (8) and Peters et al. (9). The methodology of scoping reviews follows a series of steps as follows (7): 1. Identifying the question, 2. Identifying the studies, 3. Selecting studies relevant to the review question from the results of the search, 4. Charting the data, 5. Collating, summarizing, and reporting the findings and 6. An optional consultation with relevant stakeholders. Scoping reviews start with an *a priori* protocol which describes the proposed methodology for each step. A protocol allows for transparency as to which decisions were made *a priori* or during the process of the review itself. Further details on each step of a scoping review are as follows:

1. Identifying the question
  The research question for a scoping review is often broad in nature, and is based on the specific objectives of the review. At a minimum, the review question defines the content area and scope of the review. Generally, a scoping review question will define one or two aspects that delineate the scope of the review. Perhaps the easiest approach to understand this is to compare the approach to identifying the review question to the type of question that would be appropriate for a systematic review. Systematic reviews usually are written very precisely to reflect specific key elements of a review question; for intervention questions, these are the population, intervention, comparison, and outcome (see systematic review question types, below, for further detail on key elements). Because a scoping review is describing the literature, rather extracting the study result, a scoping review about an intervention might seek to map this body of literature by defining only the population and the outcome of interest in the scoping review question. For example, while a systematic review, might ask “What the effect of BRD vaccination compared to no vaccination on the incidence of respiratory disease in feedlot cattle,” a scoping review might ask, “What interventions have been investigated for the reduction of respiratory disease in feedlot cattle?” In this example, the scoping review has defined the population and outcome, and then will map the literature about the interventions and comparators. Scoping reviews in veterinary medicine have involved a range of species and topic areas, including scoping reviews of the indicators and methods of measurement that have been used to evaluate the impact of population management interventions for dogs (10), non-antibiotic interventions in cattle to mitigate antibiotic resistance of enteric pathogens (11), and indications for acupuncture in companion animals (12).
2. Identifying the studies
  The process of searching the literature for relevant studies is the same for scoping and systematic reviews. The intention for a scoping review is to describe the totality of literature on a subject. Thus, the aim is to maximize the sensitivity of the search for identifying relevant literature. Search terms are created to address the key components of the research question, such as the population of interest and the topics area. These search terms are then combined using Boolean operators and applied to multiple electronic databases as well as other sources such as websites or theses portals (the “gray literature”). The specifics of creating and applying search strategies are consistent with those used in systematic reviews, and so this topic will be more completely covered in later sections of this article.
3. Selecting relevant studies
  The process of selecting relevant studies is the same for scoping and systematic reviews. Maximizing the sensitivity of the search generally results in a loss of specificity; many non-relevant citations may be captured. Thus, the aim of this step is to identify and remove from the review citations that are not relevant to the scoping review question. This is done by creating a small number (generally one to three) of “screening questions” that can be applied quickly to the titles and abstracts of each citation to allow the identification of citations that are not relevant. The questions often pertain to the population and outcome or topic area of interest. For instance, if the aim of the scoping review is to describe the literature on interventions to prevent respiratory vaccines in swine, the questions might ask whether the citation describes swine as the population of interest, and whether the citation describes the outcome of interest i.e., interventions to prevent respiratory disease. After screening titles and abstracts, full texts are acquired for potentially relevant citations and the screening questions are applied again to the full articles.
  To reduce the potential for selection bias in the identification of relevant literature, it is standard practice for relevance screening to be undertaken in duplicate by two reviewers working independently, with any disagreements resolved by consensus. A recent study comparing duplicate screening to limited dual review (only some of the citations screened by two reviewers) reported that up to 9.1% (title and abstract screening) and up to 11.9% (full text screening) of relevant articles were inadvertently excluded when two reviewers were not used (13). However, when the number of citations identified by the search is very large, screening can be undertaken by a single reviewer, with a second reviewer evaluating the studies which were identified as not relevant by the first reviewer. Currently, screening for relevant studies based on the title and abstract is usually conducted by human resources, however machine learning approaches are available to assist in this process, and it is envisioned this process will be fully automated soon.
4. Charting the data
  This is a step where there are substantial differences between a scoping review and a systematic review. The differences relate to the level of detail extracted and the focus; because they are descriptive, scoping reviews usually do not extract the results of a study and rarely assess the risk of bias in a study (14). For a scoping review, describing the data involves extracting relevant information from each of the articles that have been identified as relevant to the review. The actual information that is collected will depend on the intent of the review as described in the protocol, but often include characteristics of the study (such as location and year), more detailed description of the population (species, stage of production for livestock animals), and the outcomes (potentially including conceptual outcomes, operational outcomes, and outcome measurements such as incidence, prevalence, relative risk or others). Data also may be collected on the aim of each study (e.g., laboratory testing, diagnostic test development, hypothesis testing) and the study design. For example, for a scoping review to address the review question “What interventions have been investigated for the prevention of respiratory disease in swine?”, information could be extracted about the population (e.g., stage of production) and possibly further details on the outcome (e.g., identification of specific respiratory pathogens via nasal swaps vs. categorization of lung lesions at slaughter as different operational outcomes for the conceptual outcome of “respiratory disease”), although the broad descriptions of the population and outcomes of interest were already defined in the review question. It is likely that more detail would be extracted related to the interventions and comparators used, because the intent of the review was to explore that aspect of the topic. Data extraction might also include information on the type of study design, if the objective was to identify possible interventions for which there was sufficient data to conduct a systematic review.
  Data extraction is usually conducted in duplicate by two independent reviewers using a standardized form developed prior to starting the study, although this form may evolve over the conduct of a scoping review. Disagreements between reviewers are resolved by consensus or with input from a third reviewer.
5. Collating, summarizing, and reporting the results
  This step also is different from a systematic review, and does not include a meta-analysis. In this step, for a scoping review, the information extracted from each relevant article is collated and presented to the reader. This can be done using tables, figures, and text. The presentation of the information should match the objectives of the scoping study, but may include a description of the type of literature available, changes in the volume or type of literature on the topic over time, or summaries of interventions and outcomes by study design to identify areas where there may be a sufficient body of literature to conduct a systematic review. The PRISMA Extension for Scoping Reviews (PRISMA-ScR) provides guidelines for appropriate reporting of scoping studies (15).
6. Stakeholder consultation
  The sixth step, which is optional, is to include stakeholder consultation. This may occur at multiple stages of the scoping review (e.g., question formulation, identification of literature, creation of data extraction tools, interpretation of results). As an example, if the scoping review question involved a consideration of management practices at dry-off in cattle, the researchers may consider including a group of dairy veterinarians or producers when discussing the scope of the review, the search terms, and the search strategy. This could help to ensure that all relevant practices are included and that the search terms include both common and potentially less common synonyms for the various management options.
  Although this brief summary provides an overview of the steps as they are generally undertaken for scoping reviews, there is a lack of consistency in the terminology and the specific approaches used in studies referred to in the literature as “scoping studies”. Colloquially, the process of describing the literature is often called mapping or charting the literature. However, those terms are not well-defined. For example, the American Speech-language-hearing Association seems to equate the term “Evidence Map” with a systematic review (https://www.asha.org/Evidence-Maps/), while the Campbell Collaboration seems to equate the term more closely with a scoping review, describing evidence maps as a “systematic and visual presentations of the availability of rigorous evidence for a particular policy domain” (https://campbellcollaboration.org/evidence-gap-maps.html). There are two published “scoping reviews of scoping reviews” which provide details on how this methodology has been applied in the literature (14, 16) and a discussion of the issue is available by Colquhoun et al. (17). It is likely that the approach to scoping reviews will be further defined and refined over time.

### Systematic Reviews

Systematic reviews are intended to summarize the literature to address a specific question. Thus, a systematic review can be seen as an approach to compiling the results from multiple studies addressing the same research question. Detailed descriptions of the methodology as developed for human healthcare questions are available from a number of international consortiums, including the Cochrane Collaboration (18) and the Centre for Reviews and Dissemination (19). A detailed discussion of systematic reviews specific to veterinary medicine is available in a special issue of the journal “Zoonoses and Public Health” (20–25).

As with scoping reviews, the systematic review process follows specific steps. The planned approach for each of the steps is first described in a protocol, which should be completed prior to starting the actual review. Any deviations from the protocol should be acknowledged and justified in the final systematic review report or publication. This transparency allows the reader to understand which decisions were made after the review progress began and reduces the risk of biases, including outcome selection bias (26). Protocols may be published prior to starting the review on websites such as PROSPERO (https://www.crd.york.ac.uk/prospero/) or SYREAF (www.SYREAF.org), posted to University repositories, or submitted as supplementary materials with systematic review publications.

**Figure 1:**
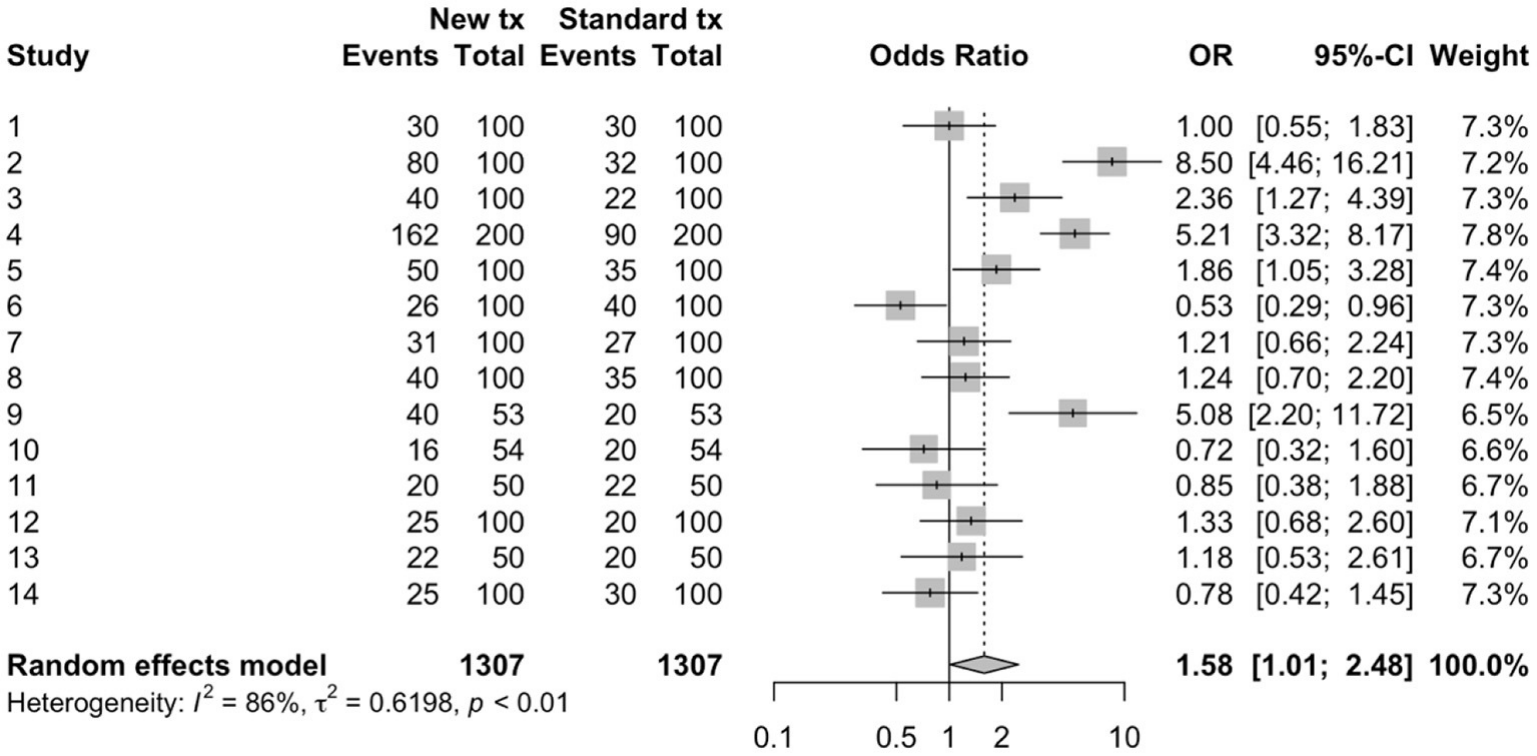
Forest plot of a meta-analysis conducted using arm-level data to estimate odds ratios from 14 hypothetical trials comparing a new treatment to a standard treatment.

**Figure 2:**
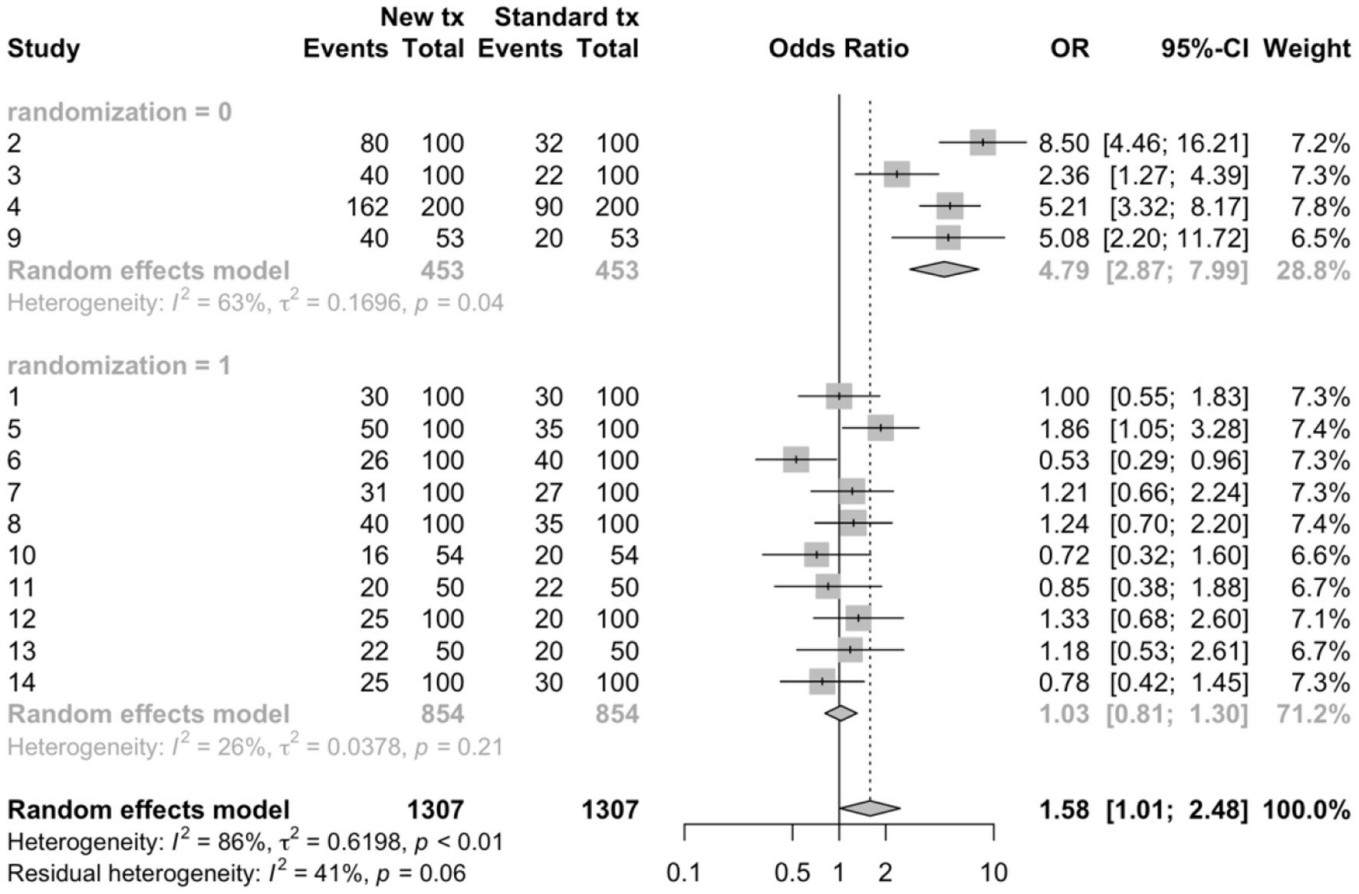
Sub-group meta-analysis to compare the results of randomized (1) vs. non-randomized (0) trials using arm-level data to estimate odds ratios from 14 hypothetical trials comparing a new treatment to a standard treatment.

**Figure 3:**
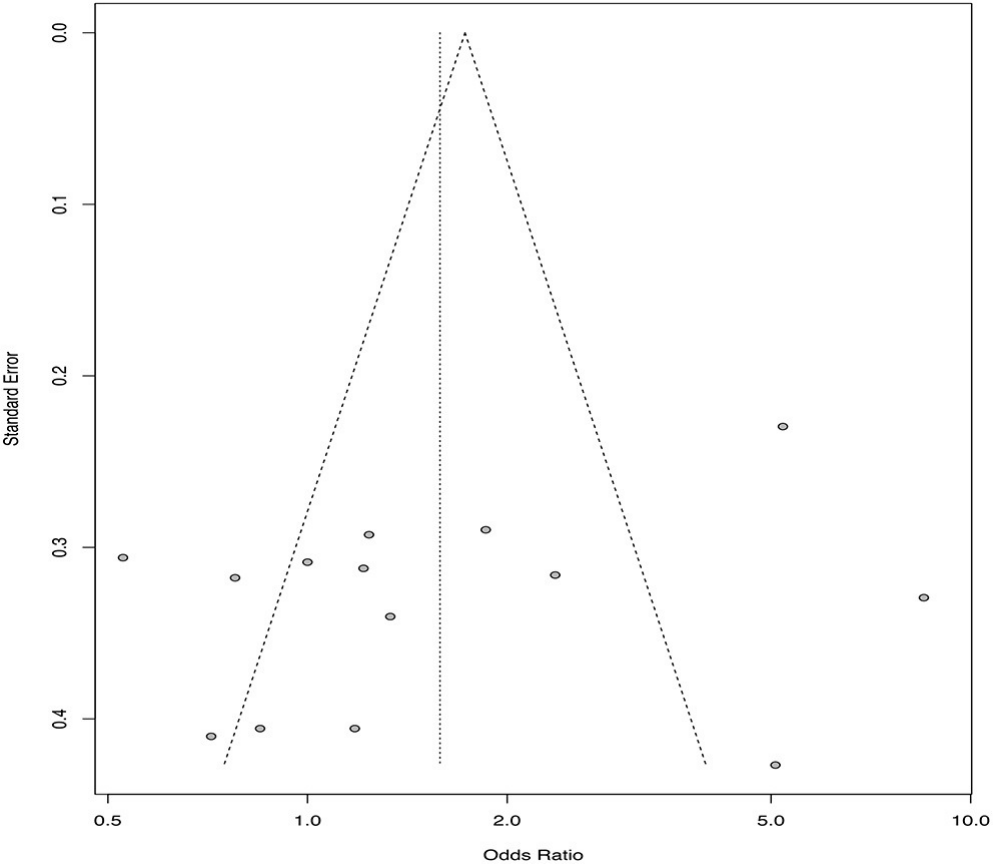
Funnel plot from 14 hypothetical trials comparing a new treatment to a standard treatment using standard error on the y-axis and odds ratio estimates from each included trial on the x-axis.

The steps of a systematic review are outlined and briefly discussed, below:

1. ***Define the review question***
  Systematic reviews can be used to address a variety of questions, but not all questions that a veterinarian might wish to have answered can be addressed by a systematic review. Generally, questions where the answer could be expressed as a list are not appropriate for a systematic review (for example: “What vaccines are available for respiratory pathogens in swine?”, or “What treatments are used in the management of FUS in cats?”), although these might be appropriate as scoping review questions. Questions that can be addressed with systematic reviews are those that could be answered with a primary research study (18, 27) where the study results estimate a parameter from a sampling distribution. To illustrate using the example of vaccines for respiratory pathogens in swine, the answer to the previously posed question “What vaccines are available?” would be a list of options. However, a related question might be “Does vaccination with *Mycoplasma hyopneumoniae* vaccines at weaning reduce the incidence of respiratory illness during the nursery stage?” For this question, the answer would be an effect size (risk ratio or odds ratio) and an associated measure of variation. Continuing the idea of combining results from multiple studies addressing the same research question, we would expect different studies to provide different estimates from an underlying sampling distribution and the goal often is to summarize the effects, report the average effect size, the observed variation in effect, and factors associated with variation in the average effect size. Some meta-analyses have different underlying assumptions and goals; for a more detailed discussion, see Rice et al. (28).
  In veterinary medicine, systematic review questions generally fall into one of four question types: descriptive questions, intervention questions, exposure or etiology questions, or diagnostic test accuracy evaluations. Each of these question types include “key elements,” which should be defined when developing a systematic review question (27).
  - *Descriptive questions*. Systematic reviews may be used to estimate parameters from a single group (effects), such as estimating incidence or prevalence of a condition, or other single group effects such as means or proportions. The key elements that need to be defined for these types of review questions are the population (P) and the outcome(s) (O). Examples of systematic reviews of descriptive questions include estimating the prevalence of *Giardia* in dogs and cats (29), the prevalence of *Campylobacter* in household pets and in petting zoos (30), and the prevalence of *Salmonella* in healthy cattle (31). Sometimes, the outcome of interest may be measured at multiple levels of organization, such as the herd level prevalence and the individual level prevalence of a disease or condition of interest, and possibly a sub-animal unit such as the quarter level in dairy cattle. These technically are different review questions; however, for efficacy, they may be combined into the same workflow process if it is expected that, by and large, the same group of manuscripts will provide the data for both outcomes. For instance, suppose that the review question of interest is “What is the prevalence of intramammary infection with *Staphylococcus aureus* in dairy cattle?” Studies addressing this question might estimate prevalence at the individual quarter level, at the cow level, and at the herd level (perhaps by sampling bulk milk or by defining a cut-point of positive samples necessary to assess a herd as positive). It would not be sensible to combine results of studies estimating prevalence at these different levels. However, if the results at all levels were of interest to the review team, it might be efficient to conduct a single search of the literature for studies estimating the prevalence of *S. aureus* in dairy cattle without specifying a level in the search terms. In this way, studies estimating prevalence at all levels would be identified, and information on the level of the study could be collected during data extraction and used to conduct separate analyses for each level.
  - *Intervention questions*. A common reason for conducting systematic reviews in veterinary medicine is to synthesize the literature evaluating the efficacy of an intervention. The key elements of this type of question are the population (P), intervention (I), comparison group (C), and outcome(s) (O); thus, review questions for interventions are often referred to by the acronym PICO (or PICOS, if the study design also is identified as a component of the review question). Examples of systematic reviews addressing intervention questions include the efficacy of porcine Circovirus type 2 vaccines in piglets (32), surgical treatments for cranial cruciate ligament disease in dogs (33), and veterinary homeopathy (34). Intervention studies usually report a metric of intervention effect compared across groups such as an odds ratio, hazard ratio, risk ratio, mean difference, or standardized mean difference. As with descriptive questions, some reviews may have multiple outcomes of interest for the same question, and these can be combined into a single review workflow. For instance, a review of the efficacy of vaccines for respiratory disease in calves may include both an outcome related to clinical disease (e.g., the relative risk of treatment with an antibiotic) and a production outcome (e.g., the mean difference in average daily gain between treatment groups).
  - *Exposure questions*. Systematic reviews also can be used to address questions related to etiology or exposures (including dose-response), with the key elements for these types of questions being the population (P), exposure (E), comparison (C), and outcome(s) (O). Examples of exposure review questions include risk factors for *Salmonella* in laying hens (35) and risk factors associated with transmission of *Mycobacterium avium subsp. paratuberculosis* to dairy calves (36). Exposure studies usually report a metric of intervention effect compared across groups such as an odds ratio, hazard ratio, risk ratio, mean difference, or standardized mean difference.
  - *Diagnostic test accuracy questions*. Systematic reviews may be used to synthesis the available literature to determine diagnostic test accuracy. The key elements of this type of review question are the population (P), index test (I), and target condition (T). Recent examples of diagnostic test accuracy systematic review include reviews to estimate the diagnostic accuracy for detecting bovine respiratory disease in feedlot cattle (37) and to compare diagnostic tests for reproductive tract infections and inflammation in dairy cows (38). Diagnostic test accuracy review questions often report metrics of test performance such as sensitivity and specificity or likelihood ratios.
2. ***Conduct a comprehensive search for studies***
  As with literature searches for scoping reviews, the aim is to identify all of the available literature on the topic. Once the systematic review question has been defined, a list of search terms is created using some or all of the PICO elements (for intervention questions), including their synonyms or other related words. The words are then combined using Boolean operators such as “AND,” “OR,” or “NOT” to create search strings (39). Filters can be applied to limit the search by year of publication, language of publication, publication type, or study design. Search strings are then applied using a search strategy to identify potentially relevant studies. Searching the veterinary literature can be challenging, in that search filters used in human healthcare literature searches are lacking for veterinary medicine and reporting of key features in titles and abstracts may be poor (40).
  A simple example of a search string as applied in Medline via Pubmed is provided for the review question “What is the efficacy of probiotics compared to no treatment for reducing or preventing diarrhea in horses” (Table 1). Key words are included for the population (horse), intervention (probiotics) and the outcome (diarrhea). Within each of these key element concepts, search terms are linked with “OR,” meaning that eligible citations need only include one of these words to be identified by the search. The key element concepts are linked with “AND,” meaning that the citations need to include at least one word in each of the key concept blocks. In this example, we have included plural forms and words; however, different databases will have symbols which allow truncation of words, as well as other features to enhance the search process. Additionally, this example is quite simplistic; key words may have been missed, and the actual syntax of the search string will differ between databases. Because of the complexity of literature searching, and the importance of identifying all of the relevant literature, including a library scientist on the review team to assist with the development of the search string and the application of the search strategy can be extremely helpful.
  Searches should be designed to capture both journal articles indexed in electronic databases and other types of research reports such as theses, government reports, and conference proceedings (referred to as the “gray literature”). It is recommended that the search include multiple databases. The electronic databases that are appropriate for systematic reviews in veterinary medicine may differ from those in human healthcare. Grindlay et al. (41) evaluated available electronic databases for coverage of veterinary journals and found that the highest journal coverage was for the Scopus database and the Cambridge Agricultural and Biological Abstracts Index. Searching the gray literature can be challenging, and the assistance of a library scientist is recommended. A recent discussion of gray literature searching, including a list of resources for searching the gray literature, is provided by Paez (42). The usefulness of available resources for searching the gray literature specifically to identify veterinary research has not been evaluated. Another approach (not mutually exclusive) is to search the reference list of recent review articles or of articles identified as relevant to the review.
  Once the searches have been conducted, the citations identified by the search are uploaded into a reference management software. Because of overlap in journal coverage between databases, there will likely be duplication citations identified by the search. Most reference management software will have an internal program that can be used to identify and remove duplicate records.
3. ***Select relevant studies from the search***
  Systematic literature searches, as with scoping review searches, are designed to maximize the sensitivity in identifying the literature of relevance to the review questions. However, this means that the specificity is often low. Thus, citations must be screened to ensure that they meet eligibility criteria for the review. The process of eligibility (or “relevance”) screening follows the same procedures as was described previously for a scoping review.
4. ***Collect data from relevant studies***
  Information of relevance to the review question is extracted from each study. This includes information at the study level such as year of publication, months, and years when the study was undertaken, study design (if multiple study designs are eligible), and geographic region. Information on the study population and, for PICO questions, the intervention and comparison groups may be collected for two reasons; they provide the necessary context for the reader to interpret the results, and they may be evaluated as possible explanations for any differences identified among the included studies. Determining *a priori* what information on the population and the intervention groups is of interest can be challenging, and it is helpful to have someone with content expertise on the review team to assist with these decisions. As with all aspects of a systematic review, these decisions should be made during the protocol development stage.
  Information related to the results of the study (or, for intervention studies, each comparison) also needs to be extracted. This includes the characteristics of the outcomes (e.g., the case definition, the method of assessing the outcome) and the result; generally, the sample size and effect in each group or the effect size, sample size, and a measure of variability. It is common for studies in the veterinary sciences to report a large number of outcomes; in clinical trials in small companion animal populations, the mean number of outcomes per trial was 10.8 and ranged from 1 to 30 (43), and in clinical trials in livestock, the mean number and range of outcomes was 8.5 and 1–41, respectively (44). Therefore, during the protocol development stage, the investigators need to decide which outcomes to use, and how many to include. Although there is no set rule, the selected outcomes should be those of relevance to decision-making and could pertain to possible harms as well as benefits. Including too few outcomes may not provide the decision-maker with enough information to make an evidence-based decision; but including too many outcomes is time intensive and may lead to a lack of focus in the review. The Grading of Recommendations, Assessment, Development and Evaluation (GRADE) working groups recommends including up to seven critical outcomes in their summary of findings tables for interpreting the quality of evidence from a systematic review (45).
  To reduce the potential for misclassification, data extraction should be conducted by two reviewers working independently with disagreements resolved by consensus, or by a single reviewer with a second reviewer validating the information extracted by comparing to the full text report, or a single reviewer after a period of duplicate extraction with verification of consistency.
5. ***Assess risk of bias in relevant studies***
  Assessing the risk of bias in the primary studies that are included in a systematic review allows for a consideration of bias in the interpretation of the results, and is a step not normally included in scoping reviews. At this stage of the review, it is not appropriate to remove studies from a review based on the risk of bias, unless one or more trial design features are included *a priori* in the eligibility criteria (for instance, eligible study designs were defined as trials with random allocation to treatment groups). However, understanding the potential for bias helps to interpret the quality of the evidence produced by a review, and factors related to risk of bias may be explored in a meta-regression or sub-group meta-analysis as possible reasons for differences in results among included trials.
  The actual criteria used in assessing bias will differ by study design. Validated tools for assessing risk of bias are available for different study designs (see: https://www.riskofbias.info/). A commonly used instrument for assessing the risk of bias in clinical trials is the Cochrane Risk-of-Bias 2.0 tool (46). In this tool, the risk of bias is assessed in five domains using signaling questions to assist the reviewer in determining a judgement for each domain. The signaling questions within each domain are answered as yes, probably yes, no, probably no, or no information. These signaling questions have been designed to make the judgement about risk of bias more consistent and reproducible (46). For each domain, an algorithm is then applied using the answers to the signaling question to determine whether the risk of bias for that domain is high, some concerns, or low. The domains relate to the risk of bias arising from the randomization process, the risk of bias due to deviations from the intended interventions, the risk of bias due to missing outcome data, the risk of bias due to the measurement of the outcome, and the risk of bias in the selection of the reported result. An overall risk of bias for the trial can then be assessed as low, some concerns or high. Some modifications to the tool may be necessary for evaluating trials conducted in some livestock populations. For instance, when assessing the risk of bias in trials in swine populations, Moura et al. (47) did not include allocation concealment in their algorithm, because the authors felt that this was unlikely to be an essential design feature for populations where all eligible pens were allocated to groups, with no reason for any *a priori* preference as to treatment group.
  Other tools are available for assessing the risk of bias for observational studies. The ROBINS-I tool was developed for assessing the risk of bias in non-randomized studies of interventions (48). Robins-I is designed for exposures that could be randomly allocated “based on a hypothetical pragmatic randomized trial.” Such a “target” trial need not be feasible or ethical: for example, it could compare individuals who were and were not assigned to start smoking. For some exposures, such as sex, age, region, or production stage, this hypothetical trial concept is not applicable; therefore, Robins-I is easiest to use when the intervention could actually theoretically be randomly allocated. Robins-I also uses signaling questions to aid the reviewer in the assessment of risk of bias, but across seven domains of relevance to non-randomized studies (48). The domains of bias assessed at the pre-intervention stage are bias due to confounding and bias due to selection of participants into the study, at the intervention stage the potential for bias due to classification of the intervention is assessed, and at the post-intervention stages the domains assessed are bias due to deviations from intended interventions, bias due to missing data, bias in measurement of outcomes, and bias in the selection of the reported results. As with the Cochrane Risk-of-Bias 2.0 tool, an overall risk of bias determination is made based on the risk of bias across the seven domains. Other tools are available for assessing the risk of bias in observational studies, such as the Newcastle-Ottawa quality assessment scale (49) and the RTI item bank (50). For systematic reviews of descriptive questions, a modification of the Quality Assessment of Diagnostic Accuracy Studies 2 (QUADAS-2) tool has been proposed for assessing the risk of bias of primary descriptive studies (51). For exposure that cannot be randomized, risk of bias tools still require validation.
6. ***Synthesize the results***
  Systematic reviews may include a qualitative synthesis or a quantitative synthesis of the results of the primary studies that were included in the review. If the studies are too disparate to justify combining then to a common result, it is still of value to present the results of the eligible studies qualitatively using tables and text. Meta-analysis provides a weighted average of the results of the individual studies (18). For intervention (PICO) questions, the results of a study are a comparison of two groups. These comparative measures are often referred to in a non-specific manner as effect sizes, a terminology that arises from clinical trials where, due to random allocation, the difference in groups is interfered as the effect of the intervention. The term effect size refers to any measure used to compare two groups. Common measures are the odds ratio, risk ratio, mean differences, and correlation. It is also possible to conduct a meta-analysis on diagnostic test evaluations. The measures used in these studies differ from group comparisons and include sensitivity, specificity, correlation, and the ROC curve. The results of several descriptive studies can also be combined as a weighted average. Common measures summarized across multiple descriptive studies include prevalence or incidence or dose response.
  The common components of a meta-analysis are the calculation of a summary effect or effect size, an evaluation of the heterogeneity of results (differences in the effect or effect size among studies), a visual presentation of these results using a forest plot, and an evaluation of the potential for small study effects (“publication bias”). Each of these is described briefly below using hypothetical data examining the odds ratio for “clinical cure” (measured as a binary variable) associated with a new intervention compared to the existing standard of care. An example dataset and R-code to calculate the various components of the example meta-analysis is included as Appendix 1 in the Supplementary Material. The interested reader can find additional details on the methodology underlying meta-analysis in Higgins and Green (18), CRD (19), and O'Connor et al. (25). We present the results using odds ratios as the outcome measure, which were calculated from arm-level data. Sometimes data are present in a study as a comparative measure, such as unadjusted or adjusted odds ratios, as opposed to arm level data. If the meta-analysis contains studies with both arm level and adjusted odds ratio, it will be necessary to convert the arm level data to the odds ratio scale because it is not possible to convert an adjusted odds ratio back to arm level data. For a meta-analysis, the data from all included studies need to be in the same form i.e., either all arm level data or all odds ratios with a measure of variability.
  Meta-analysis of binary data from two groups is usually conducted on the log odds scale (i.e., the difference in the log odds). The software package recognizes if the data are arm level or contrast level and conducts this conversion. After the analysis is complete, the data are usually converted back to the odds ratio and sometimes back to the risk ratio using the expit formula (inverse of the logit function). The presentation of risk ratio results based on back transformation of log(odds) is quite different from direct meta-analysis of the log(risk ratio) which is less common because the risk ratio has mathematical constraints that can create bias in the meta-analysis [see Bakbergenuly et al. (52)] for a discussion of this topic. Although meta-analysis of binary data from two groups is usually conducted using the odds ratio as the metric, the issue of non-collapsibility remains in meta-analysis as it does with primary research [see Rothman et al. (53) for a discussion of this topic in the primary research and Bakbergenuly et al. (52) for a discussion of this topic in meta-analysis].
  The first step of a meta-analysis is to calculate a weighted average of the effect size and then convert that, if necessary, back to a scale of interest. In our example, the meta-analysis calculates the average weighted log odds ratio and then converts that to a summary odds ratio. A meta-analysis can either calculate the weighted mean effect size using a fixed effect(s) approach [where it is assumed that the true effect size is a single common value or several single effects (28)] or using a random effects approach (where it is assumed that the true effect size follows a distribution). A random effects approach is usually considered most appropriate in the situation where the observed studies are considered to be a representative sample of the population. The fixed effect(s) approach is appropriate if the goal is to make inference conditional on the observed studies (54). Further, if the difference in effects observed in studies are not regarded as random, then a fixed effect(s) approach to analysis may be suitable. Rice et al. (28) also discusses the two underlying data generating mechanisms that can be used to make inference from a fixed effect(s) approach. The first is that there is a single true effect and sampling error causes observed differences in the estimates. This approach is discussed in detail by Borenstein et al. (55), and is referred to as the fixed effect (note singular) approach. However, Rice et al. (28) have recently proposed referring to this as a common effect model (54). The second hypothesized data generating mechanism under the fixed effect(s) approach is that the studies are estimating different effects, i.e., the variation is due to different effects and is not random. This is referred to by Rice et al. (28) as a fixed effects (note plural) approach. There has been debate over the relative merits of fixed(s) and random-effects approaches to meta-analysis and there is no consensus as to which approach is appropriate and under what circumstances (28).
  With a fixed effect approach, studies are frequently weighted based on the inverse of their variance; thus, larger studies tend to contribute more to the summary effect size than smaller studies (55). Another approach to fixed effects meta-analysis is the Mantel-Haenszel, which is used when data are sparse and requires a different weighting approach based on the summary statistics and in particular uses a weighted odds ratio rather than a log odds. The Peto method of fixed effect meta-analysis, also called the one-step method, uses the log odds ratio and a variant of the inverse variance weighting approach (55). For the random effects approach, both the within study variance and the between study variance are considered in the weighting. The method of DerSimonian and Laird (56) is a commonly used method to estimate the between and within study variance because it is the default approach in many software packages. However, there are alternative approaches to estimation that are may be better and are available in many packages (57). In the meta-analysis section of the Cochrane Handbook (58), it has been proposed that the approaches by Hartung and Knapp (59) and Sidik and Jonkman (60) should be used if available to review authors as the confidence intervals are correctly adjusted to account for the uncertainty associated with the estimation of the between study variance.
  The results of a meta-analysis often are displayed using a forest plot (Figure 1). In this plot, the results of each study are shown both numerically (columns on the right of the figure) and graphically. In the figure, the results of each comparison are shown by a box (representing the point estimate of the effect size) and by a horizontal line (representing the 95% confidence intervals). On the right of the graph, the weighting of each comparison to the final summary effect size is shown. At the bottom of the plot, the summary effect size is shown numerically (point estimate and 95% confidence intervals) and graphically, using a diamond, with the center of the diamond representing the point estimate and the horizontal ends of the diamond representing the 95% confidence intervals on the estimated mean summary effect size. In the example dataset, and using a random effects approach, the summary odds ratio was 1.58 (95% confidence intervals: 1.01, 2.48). As with primary research, the confidence interval relates to uncertainty around the average effect of the distribution and does not describe the variation in the underlying distribution of the “random effect.” Tau squared is the between study variance and the square root of tau squared, tau, is the estimate of the standard deviation across the studies. If it is of interest to describe to the reader the distribution of the studies (i.e., how much study effects vary) this can be reported by using a prediction interval. The prediction interval incorporates two levels of variation, the standard error of the estimated weighted mean of the distribution and the estimate of between study variance Tau squared (61).
  Visually assessing the forest plot in Figure 1, it is apparent that not all of the individual studies observed the same results; in some of the studies, it appeared that the new treatment was better and in some worse, and for some of the studies the 95% confidence intervals included the null value (odds ratio of 1) whereas in other studies it did not. There are two common measures used to quantify heterogeneity in the results of a meta-analysis. The first of these is Cochran's Q statistic and corresponding Chi-square based test which evaluates the homogeneity of the effect size of the studies (62). While informative, the Cochrans Q test tends to be of low power, as the number of studies included in most meta-analyses is quite low. The second measure is *I*^2^ which tells us the relationship between the two sources of variation that we expect in a meta-analysis—the variation of true effects and the variation due to sampling error (62). As such, *I*^2^ describes the percentage of total variation across studies that is due to heterogeneity in effects rather than chance. *I*^2^ is frequently reported as describing how much the effects vary. However, *I*^2^ is a proportion rather than an absolute value. If the *I*^2^ percentage is small, this implies that if all the sampling error was removed, the true effects would not differ greatly i.e., consistent true effects. If the *I*^2^ percentage is large, this implies that if all the sampling error was removed, the true effects would differ greatly among studies i.e., more variation due to true effects (62). Guidelines are available to interpret *I*^2^; values of 0–40% are likely unimportant, 30–60% represents moderate heterogeneity, 50–90% represents substantial heterogeneity, and 75–100% represents considerable heterogeneity (18). In the example meta-analysis, the *I*^2^ shows that 86% of the variability in the individual study results were due to heterogeneity, rather than chance. Thus, quantifying the summary effect size might not be useful for this example, and exploration of factors that contribute to the heterogeneity might enable better understand the effect size and factors impacting the effect size.
  Beyond sampling error, heterogeneity may be related to clinical (contextual) or methodological factors. Clinical heterogeneity results from variability in the population, intervention, or outcome, whereas methodological heterogeneity results from variability in study design or risk of bias among studies (18). Techniques are available to explore possible sources of heterogeneity. Subgroup meta-analysis can be used to explore suspected sources of heterogeneity by dividing the primary studies into subgroups based on the characteristic that is thought to be a source of heterogeneity (18). These potential sources of heterogeneity may be clinical or methodological. In the working example, a subgroup meta-analysis was conducted based on whether or not the study employed random allocation of study subjects to treatment group (Figure 2). In this figure, we can view the meta-analysis (and resulting evaluations of heterogeneity) separately for trials that used random allocation to treatment group (the lower forest plot) and those that did not (the upper forest plot). In this hypothetical example, we see that the results differ; the meta-analysis of non-randomized trials showed a large beneficial effect of the new treatment vs. the standard treatment (summary OR = 4.79, 95% CI = 2.87, 7.99), although the *I*^2^ value was still high at 63%. However, for the randomized studies, the point estimate of the summary OR suggests no benefit (1.04) and the 95% confidence interval is quite precise (0.81–1.30) suggesting that there is no evidence, on average, of benefit to the new treatment. In the meta-analyses of randomized studies, the *I*^2^ value indicates that heterogeneity was not a concern.
  Another approach to exploring heterogeneity is meta-regression. Meta-regression is a weighted regression of the results of the individual studies (the unit of concern) on the variables of interest, which often are possible sources of heterogeneity. As with meta-analysis, the weighting generally is the inverse variance of each study's result. Details on how to conduct univariable or multivariable meta-regression can be found elsewhere (18, 25).
  Publication bias is a potential concern whenever research results are considered in decision-making. Publication bias occurs because studies showing preferred results are more likely to be published or are published faster (63). We use the term “preferred result” rather than positive or negative result, as these terms can be misleading and have multiple meanings. For example, for an outcome such as mean difference, the results can be positive or negative, but depending upon the outcome, a negative or positive mean difference might be preferred. Another use of the term positive or negative might be to confer inference; for example, a vaccine a product intended to present a specific disease that has an OR greater than one compared to an untreated control has a “positive” result in absolute terms. If the outcome was mortality, a “positive” OR would not be the preferred outcome. However, if the outcome was the probability of sero-converting to the disease agent of interest, then an OR of greater than one would be the preferred outcome.
  There is empirical evidence that many research studies in the veterinary sciences are not published in the peer-review literature; a study of conference proceedings abstracts for swine and cattle vaccine trials found that <10% of the studies were subsequently published (64), and interventions related to on-farm and abattoir food safety reported that less than half of the research was published in the peer-reviewed literature within 4 years (65). Small study effects, one explanation for which is publication bias, can be assessed using a funnel plot, which plots the effect size from individual studies on the x-axis and some measure of variability (inverse variance, standard error, inverse of the standard error, or sample size) on the y-axis (63, 66, 67). The resulting figure is called a funnel plot because the precision of an effect size increases as the sample size increases. Thus, it would be expected that smaller studies would have a wider range of estimates and larger studies a smaller range of estimates, leading to a funnel shaped plot in the absence of publication bias. Publication bias will result in an asymmetric shape, with smaller non-significant publications not represented, although it should be noted that there are reasons other than publication bias that may result in a non-symmetric funnel (67). An example might be if both challenge studies and natural disease exposure trials are included in the same review; challenge studies tend to be smaller and also tend to report a larger effect size (68). This is an example of a source of heterogeneity whereby small studies may have a different effect compared to larger studies. Funnel plots represent a visual approach to detecting publication bias, although it may be difficult to accurately assess whether publication bias is present or not based on a visual appraisal (69). There are statistical tests available to formally evaluate asymmetry in a funnel plot, the most common being a rank correlation test and the regression test (63, 70, 71). Other approaches to detecting publication bias include the selection model approach. Selection models use the weighted distribution theory to model the selection and can be complicated, especially compared to the funnel plot approach. A comprehensive review of this approach is available (72). In addition to detecting publication bias, it might be interest to quantify the effect of publication bias on the effect size and adjust for the bias. A review of these methods is available elsewhere (73). A funnel plot for the example data using standard error as the measure of variability on the y-axis is shown in Figure 3.
7. ***Present the results***
  The results of a meta-analysis generally are presented using text, figures, and tables. In most cases, the presentation of results will include a table summarizing the study characteristics, as well as tables and figures showing the individual study results and the results of risk of bias assessments. If a formal meta-analysis was conducted, forest plots and funnel plots also may be shown. A summary of the findings of a systematic review by outcome may be presented in a “summary of findings” table (45). These tables include the summary effect size with 95% confidence intervals, the total number of participants and studies, an estimate of absolute effect, and the overall quality of evidence (see below).
  The methods and results of a systematic reviews should be reported in sufficient detail that the reader can evaluate the potential for bias. The Preferred Reporting of Items for Systematic Reviews and Meta-analyses (PRISMA) guidelines provide recommendations for the items of information and the level of detail that should be included in a systematic review report [(74, 75); www.prisma-statement.org].
8. ***Interpret the results***
  If one or more meta-analyses are conducted, the results should be interpreted in the context of the magnitude of effect and the confidence in the evidence (quality of evidence). The magnitude of effect relates to the summary effect size and its variability. One approach to evaluating the quality of evidence is by using the Grading of Recommendations, Assessment, Development and Evaluation (GRADE) (76, 77). The overall quality of evidence is determined to be high, medium, low, or very low. The framework evaluates the quality of evidence by considering four domains; risk of bias, publication bias, imprecision, inconsistency, and indirectness. If there are serious concerns in a domain, the overall quality of evidence will be downgraded by one level; if the concerns are very serious within a domain, the quality of evidence can be downgraded by two levels. Reviews of randomized trials start at the rating of “high,” whereas observational studies start at a level of “low.” Observational studies may be upgraded, as well as downgraded. The domains for evaluating quality of evidence, with references to further details for each, are as follows:
  - *Risk of bias* (78). In this domain, the risk of bias in the individual studies is considered. If most of the evidence is from studies with a high risk of bias, the reviewer may wish to downgrade the quality of evidence. For instance, if the majority of evidence in a review came from trials where allocation to treatment group was not random, the quality of evidence would be lower than a review where the individual trials employed random allocation to treatment group.
  - *Publication bias* (79). If there is strong evidence of publication bias, to the point where the reviewer believes that it may have impacted the review results, the quality of evidence may be downgraded.
  - *Imprecision* (80). Imprecision may be a concern if the overall sample size is less that the sample size that would be appropriate to address the research question in a single study, or if the confidence intervals on the summary effect size span harm, no association, and benefit.
  - *Inconsistency* (81). Inconsistency is related to the heterogeneity in the results. If there was considerable heterogeneity in the results, but the reviewers were able to explain the sources of that heterogeneity (for instance, using subgroup meta-analysis), then inconsistency may not be a concern. However, if there is substantial unexplained heterogeneity, then the quality of evidence may be downgraded.
  - *Indirectness* (82). Indirectness relates to the applicability of the evidence to the research question. Indirectness may relate to one or more of the PICO elements. For instance, if the question of interest was the efficacy of a treatment in market weight pigs, and yet most of the studies identified evaluated the treatment at the start of the finishing period, the evidence would be less direct.
  Examples of the use of GRADE in veterinary systematic reviews include a review of furosemide for exercise-induced pulmonary hemorrhage in racehorses (83), a review of the of the efficacy of whole-cell killed *Tritrichomonas foetus* vaccines in beef cattle (84), and a review of on-farm interventions to reduce *Salmonella* in swine (85).

**Table 1:** Example of a simple search strategy that could be used to identify studies evaluating the efficacy of probiotics to reduce or prevent diarrhea in horses using Medline via PubMed.

**Search**	**Query**	**Items found**
#1	Horse or horses or pony or ponies or donkey or donkey or equine	98,264
#2	Probiotic or probiotics or yeast or lactobacillus or “lactic acid bacteria” or bifidobacteria or Saccharomyces or “*Bacillus subtilis*”	367,665
#3	Diarrhea or enteric or gastrointestinal or GI or scours	532,459
#4	#1 and #2 and #3	60

## Future Directions

There are a number of exciting enhancements to systematic reviews that are recently available or are likely to be available in the new future. These include automation of some of the steps of a systematic review, “living systematic reviews” which evolve as new information becomes available, and network meta-analysis, where multiple intervention options for the same outcome can be assessed in a single analysis which includes both direct and indirect evidence.

Systematic reviews not only are time sensitive, but also require a considerable input of time and resources, taking an average of 67 weeks from protocol registration to publication (86). Thus, having mechanisms to semi-automate at least some stages of a review using machine learning or natural language processing is inherently appealing. Recently, the journal “Systematic Reviews” published an editorial and three commentaries outlining the state of automation in systematic reviews (87–90). Existing tools for using automation in systematic reviews are available and may be used to identify randomized controlled trials, or for eligibility screening, data extraction, or risk of bias. However, there is a need to validate existing tools and continue to develop the techniques. This is a rapidly developing area in systematic reviews and it is likely that automation of some steps of a systematic review will become more common over time.

Systematic reviews provide a rigorous method of synthesizing the literature, but they represent the evidence at a static point in time. Research is identified up until the date that the literature search was conducted. However, it then takes time to conduct the review and for the review to make its way through the publication process. In human healthcare, it has been estimated that reviews are seldom updated within 2 years of publication (91). Thus, the information available in published systematic reviews may not represent the current state of knowledge (92). Nonetheless, updating systematic reviews requires time and resources, and periodic updating does not negate the time between the conduct of a literature search and formal publication (92). However, technological advances, including automation, allow for the creation of living systematic reviews (92). Unlike traditional systematic reviews, which are in the form of written reports or publications, living systematic reviews are dynamic on-line evidence summaries which can be updated frequently and rapidly as new information becomes available in the literature (92). As the technology and approach become more common, living systematic reviews have the potential to provide the most up to date and rigorous summary of the evidence possible to assist veterinarians and producers in making the most evidence-informed clinical decisions possible.

## Data Availability Statement

The raw data supporting the conclusions of this article will be made available by the authors, without undue reservation, to any qualified researcher.

## Author Contributions

JS and AO'C co-determined the content and scope of the review. JS drafted the manuscript. AO'C reviewed the manuscript. All authors approved the final contents.

### Conflict of Interest

The authors declare that the research was conducted in the absence of any commercial or financial relationships that could be construed as a potential conflict of interest.
