# Supplementary material for: Scoping Reviews, Systematic Reviews, and Meta-Analysis: Applications in Veterinary Medicine
Source: Front Vet Sci. 2020 Jan 28;7:11. doi: 10.3389/fvets.2020.00011 (PMC6997489; doi:10.3389/fvets.2020.00011)
Supplement: Supplementary file 1 [file Data_Sheet_1.docx]

**Appendix 1:** Dataset and R code for conducting meta-analysis of arm-level binomial data, obtaining a forest plot, and creating a funnel plot using the R package *meta* (version 4.9-6) in Rstudio (Version 1.2.1335). This code assumes the user knows how to work in the R environment. R is a free-ware available at <http://cran.r-project.org/>. R and R packages are updated frequently and the options may change. Thus, there may be differences if the user if not using the same version of the package or the software.

# Installing the necessary package in R to run a meta-analysis

install.packages("meta")

library(meta)

# Setting up the directory and files to use

setwd(dirname(file.choose()))

# Getting R to read the dataset

dat <- read.csv("example.csv")

# if you wish to see the data

View(dat)

#Coding to run a random effects meta-analysis using arm-level data to calculate odds ratios as the outcome measure, calculate measures of heterogeneity, and display a forest plot

metaOR<-metabin(event.e, n.e, event.c, n.c, studlab=trial, sm="OR", comb.fixed = F, comb.random = T, data=dat)

summary(metaOR)

forest.meta(metaOR, lab.e = "New tx", lab.c = "Standard tx")

# Setting the variable “randomization” as a categorical variable

labels(dat)

dat$randomization <- as.factor(dat$randomization)

# Running a sub-group meta-analysis based on whether or not the trial used random allocation to treatment group

meta1<-metabin(event.e, n.e, event.c, n.c, studlab=trial, sm="OR", comb.fixed = F, comb.random = T, data=dat, byvar = randomization, title="Dat")

summary(meta1)

forest.meta(meta1, lab.e = "New tx", lab.c = "Standard tx")

# evaluating small study effects via a funnel plot using standard error for the y-axis

funnel(metaOR, yaxis="se")

Dataset used to create forest plots and funnel plots.

| trial | experimental | control | event.e | noevent.e | n.e | event.c | noevent.c | n.c | randomization |
| --- | --- | --- | --- | --- | --- | --- | --- | --- | --- |
| 1 | New_tx | Old_tx | 30 | 70 | 100 | 30 | 70 | 100 | 1 |
| 2 | New_tx | Old_tx | 80 | 20 | 100 | 32 | 68 | 100 | 0 |
| 3 | New_tx | Old_tx | 40 | 60 | 100 | 22 | 78 | 100 | 0 |
| 4 | New_tx | Old_tx | 162 | 38 | 200 | 90 | 110 | 200 | 0 |
| 5 | New_tx | Old_tx | 50 | 50 | 100 | 35 | 65 | 100 | 1 |
| 6 | New_tx | Old_tx | 26 | 74 | 100 | 40 | 60 | 100 | 1 |
| 7 | New_tx | Old_tx | 31 | 69 | 100 | 27 | 73 | 100 | 1 |
| 8 | New_tx | Old_tx | 40 | 60 | 100 | 35 | 65 | 100 | 1 |
| 9 | New_tx | Old_tx | 40 | 13 | 53 | 20 | 33 | 53 | 0 |
| 10 | New_tx | Old_tx | 16 | 38 | 54 | 20 | 30 | 54 | 1 |
| 11 | New_tx | Old_tx | 20 | 30 | 50 | 22 | 28 | 50 | 1 |
| 12 | New_tx | Old_tx | 25 | 75 | 100 | 20 | 80 | 100 | 1 |
| 13 | New_tx | Old_tx | 22 | 28 | 50 | 20 | 30 | 50 | 1 |
| 14 | New_tx | Old_tx | 25 | 75 | 100 | 30 | 70 | 100 | 1 |
